# Use of Betaine-Based Gel and Its Potential Application in Enhanced Oil Recovery

**DOI:** 10.3390/gels8060351

**Published:** 2022-06-03

**Authors:** Yuman Wu, Jie Zhang, Sanbao Dong, Yongfei Li, Michal Slaný, Gang Chen

**Affiliations:** 1 Shaanxi Province Key Laboratory of Environmental Pollution Control and Reservoir Protection Technology of Oilfields, Xi’an Shiyou University, Xi’an 710065, China; 20211070832@stumail.xsyu.edu.cn (Y.W.); zhangjie@xsyu.edu.cn (J.Z.); dongsanbao@xsyu.edu.cn (S.D.); yfli@xsyu.edu.cn (Y.L.); 2 State Key Laboratory of Petroleum Pollution Control, CNPC Research Institute of Safety and Environmental Technology, Beijing 065001, China; 3 Xi’an Key Laboratory of Tight Oil (Shale Oil) Development, Xi’an Shiyou University, Xi’an 710065, China; 4 Institute of Inorganic Chemistry, Slovak Academy of Sciences, Dúbravská cesta 9, 845 36 Bratislava, Slovakia; 5 Institute of Construction and Architecture, Slovak Academy of Sciences, Dúbravská cesta 9, 845 03 Bratislava, Slovakia

**Keywords:** viscoelastic, fracturing fluid, temperature resistance, oil displacement, surfactant

## Abstract

In this paper, a betaine-based gel containing 2.0% erucamide propyl betaine (EAPB), 0.5% oleic acid amide propyl betaine (OAPB), and 0.1% KCl was prepared for use as a fracturing fluid. The performance evaluation showed that KCl may improve the temperature resistance and increase the viscosity of the optimized fracturing fluid. At 80 °C, the apparent viscosity of the viscoelastic surfactant (VES)-based fracturing fluid was approximately 50 mPa·s. Furthermore, the gel had high shear resistance, good viscosity stability, and high sand-carrying performance. After being sheared at 170 s^−1^ for 60 min, the reduction in viscosity was 13.6%. The viscosity of the gel was relatively stable at room temperature (27 °C) for one week. In a suspension containing 10% sand (particle size < 0.45 mm, density = 2.75 g cm^−3^), the settling velocity of proppant particles was 1.15 cm h^−1^. In addition, we detected that the critical micelle concentration of this gel was approximately 0.042 wt%. The viscosity could be reduced to <5 mPa·s at 60 °C within 1 h when 6.0% crude oil was present, and oil displacement experiments showed that the broken fracturing fluid can enhance the oil displacement rate up to 14.5%. This work may facilitate research on fracturing fluids and oil recovery.

## 1. Introduction

The exploitation of tight reservoirs becomes more important with the depletion of conventional high-permeability reservoirs. However, due to the low permeability nature of the tight reservoirs, extracting crude oil from these kinds of reservoirs is difficult. Hydraulic fracturing is a commonly used well stimulation technology to increase oil and gas production [1] and is regarded as the key treatment for exploiting low permeability oil and gas fields. Nowadays, fracturing fluid is an important factor affecting the efficiency of hydraulic fracturing [2]. On the other hand, there are ongoing concerns about the air, soil, and water contamination caused by hydraulic fracturing activities. Green chemicals are used in fracturing fluid to mitigate these effects [3].

However, conventional polymer fracturing fluids, such as guar gum (GG), have been used due to their ability to regulate the rheological properties and enhance the viscosity of aqueous solution. However, the swelling rate of GG is slow, and its efficiency is easily degraded by microorganisms [4]. Furthermore, GG contains plenty of water-insoluble fibers and residues after being broken down (residue content, 500 mg/L∼1000 mg/L), directly influencing the fracturing effect and damaging the formation [5,6,7]. As reported by Hang et al. [8], only 30–45% of the injected guar-based polymer fluids could return from the well during the flow-back period [9,10,11]. At the same time, compared with traditional fracturing fluids, viscoelastic surfactant (VES)-based fracturing fluids have attracted wide attention because of the advantages of high viscoelasticity, low friction, easy flow back, and environmentally friendliness [12]. The fracturing fluid’s average permeability damage to the reservoir’s natural core in the target block was 6.07% for the fracturing fluid prepared by Yang et al. [13]. This showed that the fracturing fluid system had low damage, effectively reducing the secondary damage after fracturing and playing a specific role in reservoir protection [13]. Clean fracturing fluids are divided into ionic clean fracturing fluid (cationic and anionic), nonionic clean fracturing fluid, and amphoteric clean fracturing fluid [13,14]. The VES-based fracturing fluid exhibited good viscoelasticity due to the three-dimensional network formed by the entangled wormlike micelles of the surfactants [11]. Both erucamide propyl betaine (EAPB) and erucic acid amidopropyl hydroxyl sulfobetaine (EAPS) are amphoteric surfactants with an ultra-long carbon chain (longer than C18) [15] that contain 25 hydrophobic carbons, double bonds, and amide bonds in the structure. These surfactants have high temperature resistance, shear and salt resistance, sterilization, biodegradation, and other good properties. Compared with betaine with the conventional short carbon chain, the EAPB and EAPS can spontaneously form wormlike micelles and enhance the viscoelasticity of the VES-based fracturing fluid. With the presence of additives, the properties of the fracturing fluids containing VES could be greatly increased, which could meet the basic properties of the fracturing fluid under the condition of low dosage.

**Figure 1:**
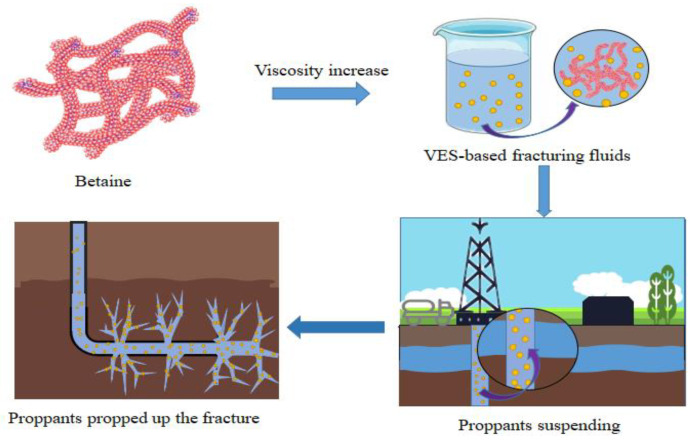
The preparation and application of VES-based fracturing fluid.

According to the above situation, the EAPB and OAPB were selected as the primary agents of a VES-based fracturing fluid in this paper. Furthermore, the development of new oil displacement technologies to improve the recovery of oil fields has become a hot issue [16], and surfactant flooding is also one of the commonly used methods in oil flooding [17]. Therefore, in this paper, a broken VES-based fracturing fluid (Figure 1) was used in an oil displacement experiment to achieve a dual-function for VES-based fracturing fluid. To the best of our knowledge, this work is new and represents progress on this issue. Moreover, our research group studied and evaluated the effects of halide anions, reaction conditions, etc. on surface-active surface tension, foaming ability, high-temperature resistance, methanol resistance, and salt resistance through compounding and synthesis methods [18,19,20,21]. In addition, finding a cost-effective crude oil flow improver to reduce the viscosity of heavy oil is another important challenge due to the low flow characteristics of heavy oil. Therefore, our laboratory has also synthesized a series of surfactants for crude oil flow improvers using surfactants and small molecules [22,23,24] such as alkylbenzenesulfonates, which have good performance in oil recovery and promising application potential [22]. The above research work is expected to expand the application scope of surfactants and provide new methods and ideas in the oil and gas fields.

## 2. Experimental

### 2.1. Materials

Oleylamine propyl betaine (40 wt%) (OAPB) was purchased from Huainan Huajun New Material Technology Co., Ltd., Beijing, China. Erucamide propyl betaine (40 wt%) (EAPB) and (40 wt%) (EAPS) were purchased from Shanghai Shengxuan Biochemical Co., Ltd., Shanghai, China. KCl (AR) was purchased from Tianjin Damao Chemical Reagent Factory in China. KBr (AR) was purchased from Tianjin Baishi Chemical Co., Ltd., Tianjin, China. KF·2H_2_O (AR) was purchased from Tianjin Chemical Reagent Factory, China and KI (AR) was purchased from Tianjin Kemio Chemical Reagent Co., Ltd., Tianjin, China. All chemicals were used as received without further purification.

### 2.2. Surfactant Screening

**Figure 2:**
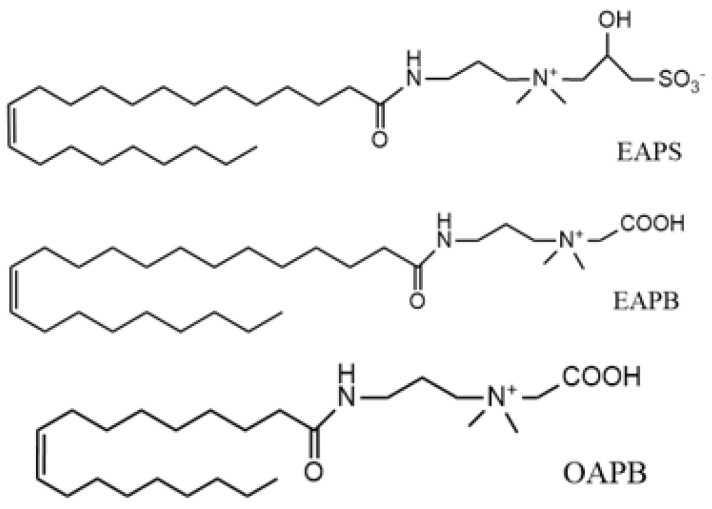
Chemical structures of the surfactants.

A six-speed rotary viscometer (MK-03, 620 × 300 × 490 mm, Beijing, China) was used to evaluate the ability of EAPB, EAPS, and OAPB to enhance the viscosity of the surfactant solution with various dosages (Figure 2).

### 2.3. Salt Screening

Based on the results of surfactant screening, the selected surfactant was mixed with each of the salts (KF·2H_2_O, KCl, KBr, and KI). The viscosity was measured at various concentrations and temperatures to find the optimized salts. After screening surfactants and salts, an optimized formula of the VES-based fracturing fluid was obtained.

### 2.4. Shear Resistance Properties Evaluation

The viscosity of the optimized VES-based fracturing fluid was measured at different conditions to evaluate the shear resistance properties. Firstly, the fracturing fluid was measured at a constant temperature and shear rate (80 °C, 170 s^−1^) for a period of time. Subsequently, it was measured at 60 °C for shear rate ranges of 170 s^−1^. Besides, the effect of constant shearing (170 s^−1^) on the viscosity of the optimized VES-based fracturing fluid would also be investigated at various temperatures. The sample was performed on a Physica MCR 301 (Anton Paar, Graz, Austria) rotational rheometer equipped with a searle-type concentric cylinder geometry CC27(ISO3219) [25].

### 2.5. Proppants Suspending Performance

In this section, a certain amount of proppant particles (particle size < 0.45 mm, density = 2.75 g cm^−3^) were mixed with the optimized fracturing fluid in a beaker and then placed into a graduated cylinder at constant temperature conditions. The settling time for the proppant to be deposited on the cylinder’s bottom was recorded as the settling velocity.

### 2.6. Gel Breaking Properties

The surface tension of each gel’s breaking fluid was measured using a tensiometer (K100, Krüss GmbH, Mainz, Germany), and the critical micelle concentration (cmc) was determined from the inflexion of the surface tension [26]. Furthermore, we used distilled water to check the tensiometer’s accuracy before experimenting. To obtain equilibrium values, all experiments were repeated at least three times [27]. A certain amount of crude oil was mixed with the VES-based fracturing fluid at 80 °C. Then, the viscosity of the VES-based fracturing fluid containing crude oil was measured to record the gel-breaking process.

### 2.7. Oil Displacement

**Figure 3:**
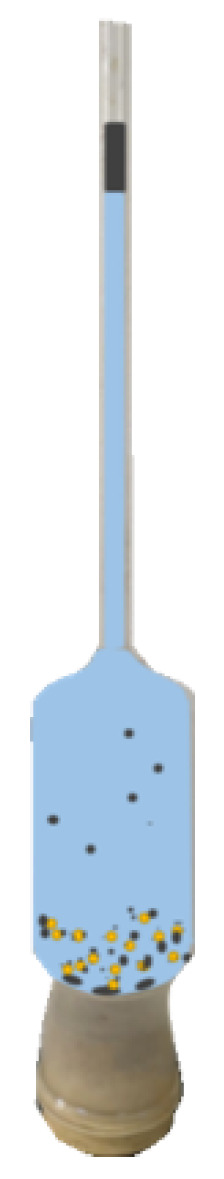
De-oil apparatus.

In this section, 200 g clean proppant was mixed with 25 g crude oil (0.82 g cm^−3^) and then placed in a desiccator at 60 °C for 18 h. Then, 8 g oil-treated sand was mixed with 135 mL fracturing fluid and placed in a de-oil apparatus at 80 °C. The experiment was performed in a water bath. The picture below shows the de-oil apparatus (Figure 3). The oil detached from the sand surfaces in the presence of fracturing fluid. The volume of the extracted oil in the upper layer of the liquid was recorded to calculate the oil extraction efficiency.

## 3. Results and Discussion

### 3.1. Mechanism of Gel Formation

**Figure 4:**
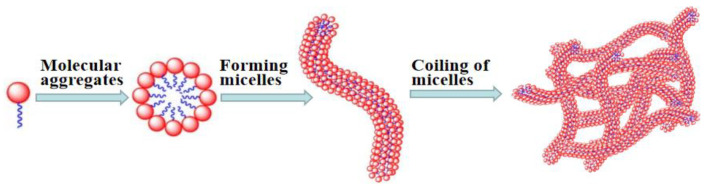
Schematic diagram of surfactant gel formation.

Each VES molecule contains a hydrophilic head-group and hydrophobic hydrocarbon chain, forming wormlike micelles through self-assembly in aqueous solution [28]. With the increase in VES concentration, wormlike micelles are entangled with each other to form a three-dimensional network structure (Figure 4) [29]. Thus, it exhibits significant viscoelastic property, facilitating the formation of viscoelastic gels.

When the inorganic salts are present, the viscoelasticity of the solution is due to the countering effect of the ions that contribute to the reduction in repulsive forces between the polar groups of VES. Thus, reducing the optimal surface group area occupied by a single head group. This enlarges the packing parameter of the surfactant molecules and promotes the surfactant micelles changing from sphere or spheroid to wormlike, cylindrical micelles, planar bilayers, and reversal micelles [30,31]. Generally, the smaller hydrate radius of the ion makes it more efficient in electrostatic screening to promote the micelle entanglement [32].

### 3.2. Formula Optimization

#### 3.2.1. Surfactant Screening

**Figure 5:**
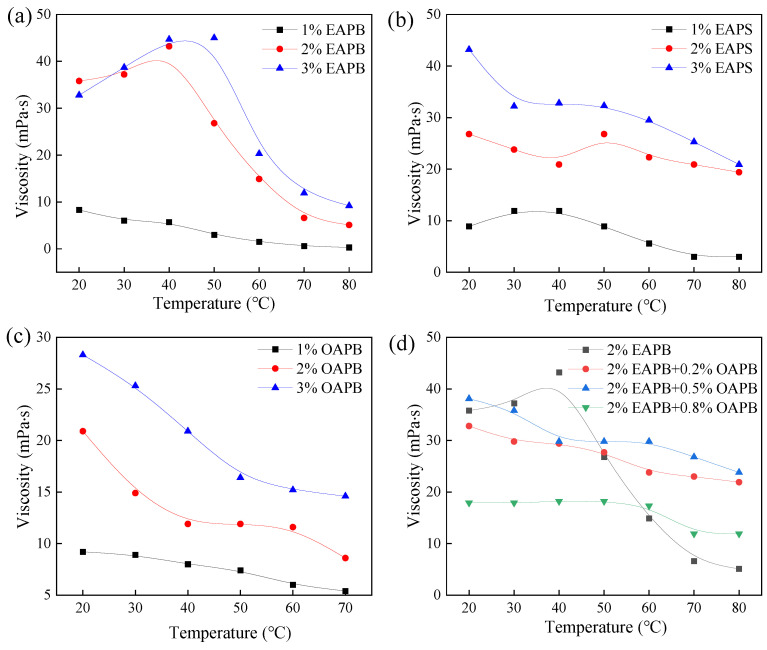
Apparent viscosity of surfactant solutions at different concentrations and temperatures: (**a**) EAPB; (**b**) EAPS; (**c**) OAPB; (**d**) EAPB + OAPB. All tested shear rates were 100 r min^−1^.

Gao et al. [28] investigated surfactant gels with potential for oilfield applications and selected surfactants with better performance by testing the viscosity of the solutions between 30 and 80 °C. The concentration of erucamide propyl betaine was 1.7%. Therefore, in this study, 1%, 2%, and 3% concentrations were selected for surfactant screening. As shown in Figure 5a, the apparent viscosity of the 1% EAPB solution decreased with the increase in temperature. For the solutions containing 2% EAPB, the viscosity increased first and then decreased with the increase of temperature, the maximum viscosity of which is 43.2 mPa·s in the range of 40–50 °C. This may be because, with the increase in temperature, the entanglement of micelles leads to a viscosity increase. However, when the temperature exceeds 50 °C, the micelles formed are destroyed, and the viscosity decreases [31].

For the solution containing 3% EAPB, a similar viscosity-temperature curve was obtained. As shown in Figure 5b, the apparent viscosity of the 2% EAPS solution increased as the concentration increased, and it exhibited good temperature resistance. The viscosity decreased from 29.8 mPa·s to 16.4 mPa·s at 20–80 °C. The maximum viscosity of the 2% EAPS solution was lower than that of the 2% EAPB solution. As shown in Figure 5c, the apparent viscosity of each OAPB solution decreased with the increase in temperature. The viscosity of the 2% OAPB solution was lower than the solution containing 2% EAPB or 2% EAPS. Therefore, 2% EAPB was used as the primary agent. Since OAPB can form micelles at a concentration of 1%, we selected OAPB below 1% to mix with EAPB. As shown in Figure 5d, with OAPB and EAPB, the viscosity was less affected by temperature. The synergy effect between OAPB and EAPB would lead to more stable wormlike micelles containing EAPB and OAPB. However, if the concentration of OAPB was higher than 0.5%, the synergy effect between EAPB and OAPB would be retarded. Therefore, the optimized dosage of OAPB was 0.5% in the presence of 2% EAPB.

#### 3.2.2. Salt Screening

**Figure 6:**
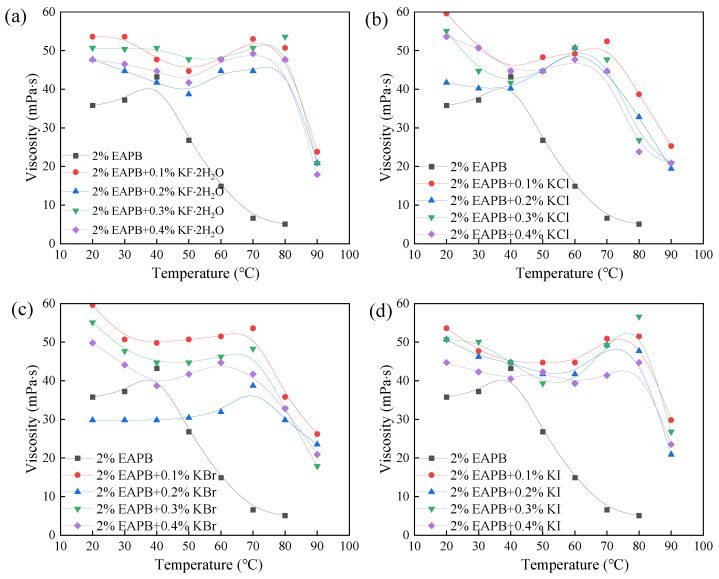
Effect of different potassium salt concentrations on viscosity (**a**) KF; (**b**) KCl; (**c**) KBr and (**d**) KI. (100 r min^−1^).

**Figure 7:**
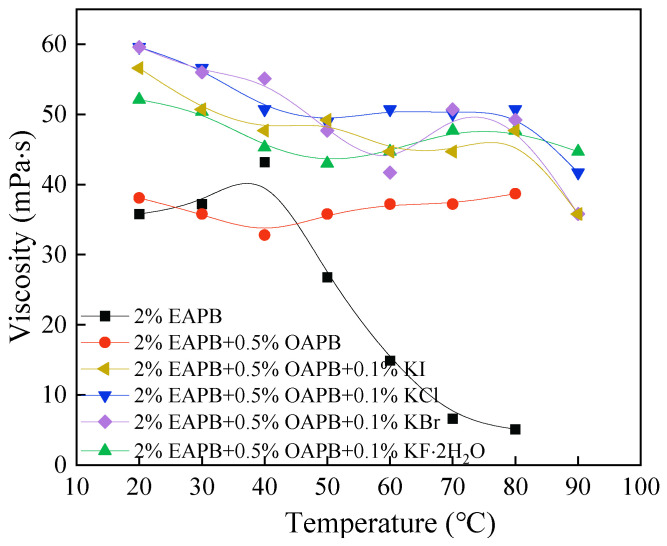
Effect of potassium salt on viscosity (100 r min^−1^).

In this section, 2% EAPB and 0.5% OAPB were mixed with other potassium halides (KF·2H_2_O, KCl, KBr, KI) to find a suitable salt to increase the viscosity of the VES solution. Figure 6 shows that the temperature resistance of the solution containing 2% EAPB + 0.5% OAPB was improved by salts, which may be due to the counter-ions from inorganic salts that promote the aggregation of VES via electrostatic interactions and reduce the repulsion between the polar head groups [30]. In addition, Figure 6 shows that the increase in salt concentration decreases the solution viscosity slightly. When the salt content was 0.1%, the solution viscosity was highest. This is probably because a further increase in salt concentration leads to the contraction of hydrophobic chains. When the contraction effect of the chains is greater than the shielding effect, the viscosity decreases [10]. As presented in Figure 7, when the temperature was >80 °C, the viscosity of each solution containing 2% EAPB, 0.5% OAPB, and salt decreased, attributed to the destruction of the micelles [31]. Besides, the solution with 2% EAPB + 0.5% OAPB + 0.1% KCl exhibited the highest viscosity-enhancing performance. For this reason, the optimized formula of the VES-based fracturing fluid was 2% EAPB + 0.5% OAPB + 0.1% KCl.

### 3.3. Viscosity Stability Test

**Figure 8:**
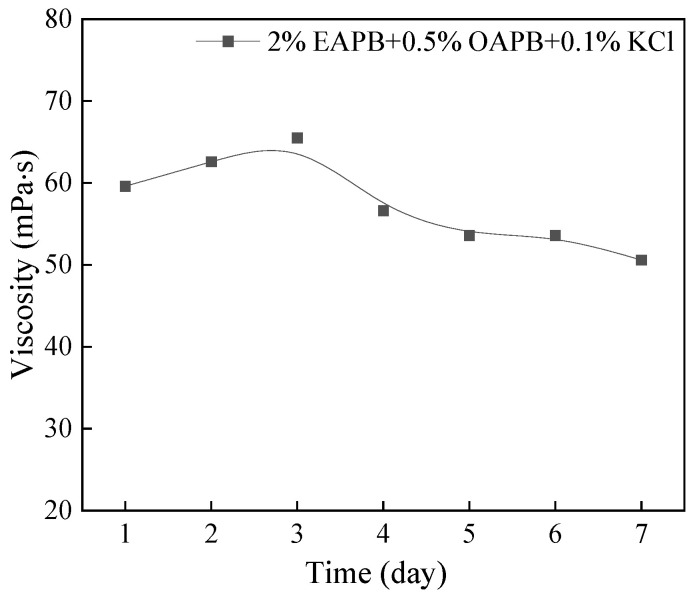
The apparent viscosity of the formulation at room temperature (27 °C) kept for one week (shear rare at 100 r min^−1^).

Viscosity stability is one of the main factors affecting the application of a fracturing fluid. In this section, the VES-based fracturing fluid containing 2% EAPB + 0.5% OAPB + 0.1% KCl was prepared, kept undisturbed for a week, and then measured to view changes in viscosity. As shown in Figure 8, the viscosity remained above 50 mPa·s, indicating that the gel was relatively stable.

### 3.4. Shear Resistance

**Figure 9:**
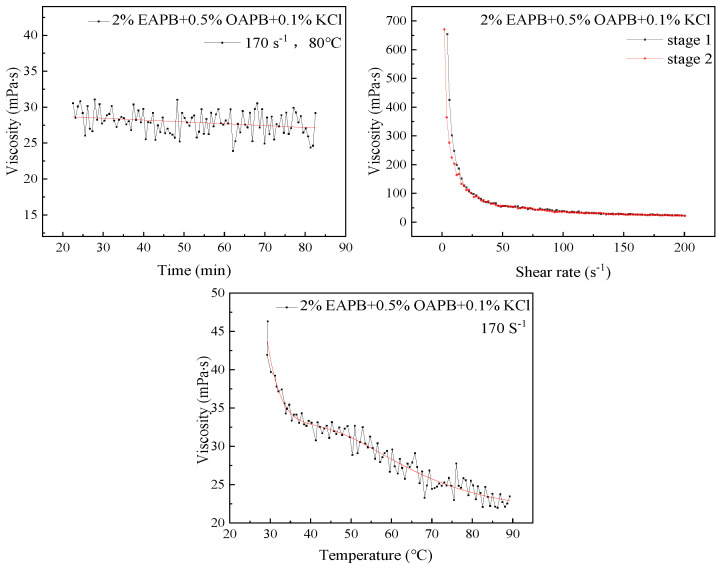
Shear resistance test.

It can be seen from Figure 9a that the viscosity of the VES-based fracturing fluid containing 2% EAPB + 0.5% OAPB + 0.1% KCl decreased with increasing shear time. After being sheared at 170 s^−1^ for 60 min, the viscosity reduction rate was 13.6%. The viscosity of this optimized VES-based fracturing fluid did not change much, indicating good shear stability. Figure 9b shows that the viscosity of the optimized VES-based fracturing fluid decreased with overall shear rate increase at 60 °C in stage 1 and then increased with decreasing shear rate in stage 2. When the shear rate was lower than 50 s^−1^, the viscosity recovered rapidly, showing that the viscosity can be restored and the micelles are reversible. Figure 9c presents the viscosity of the optimized VES-based fracturing fluid as the temperature ranges from ambient temperature to 90 °C at a shear rate of 170 s^−1^. Interestingly, the viscosity of the optimized VES-based fracturing fluid remained unchanged in the temperature range of 40–50 °C. The viscosity of the fracturing fluid decreased gradually as the temperature further increased from 50 °C to higher temperatures.

### 3.5. Viscoelastic Experiment

The viscoelasticity of clean fracturing fluid represents the comprehensive properties of elasticity and viscosity. The unique elastic behavior of clean fracturing fluid is one of the important reasons for its high sand-carrying performance even when the viscosity is relatively low. In this experiment, the viscoelastic stress interval of the VES-based fracturing fluid was determined by a stress scan at 30 °C and 1 Hz. Then, the dynamic frequency sweep test was selected in a corresponding area of the stress value (0.02 Pa).

**Figure 10:**
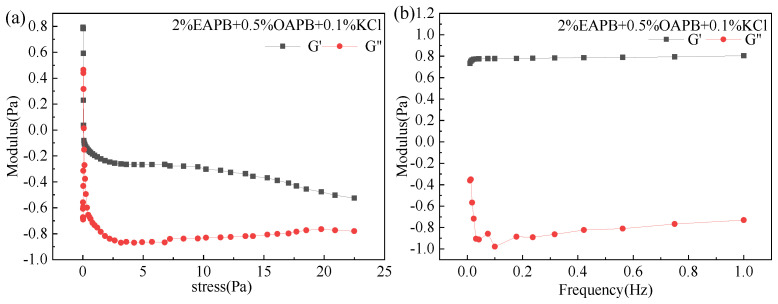
(**a**) Dynamic stress scanning results. (**b**) Dynamic frequency scanning results. (The modulus in the figure is taken as the value after l g).

According to Figure 10a, the storage (G′) and loss (G″) modulus have no relationship with the change of stress within a specific stress range, so there is a linear viscoelastic region. When the external force is greater than a certain value, the elasticity decreases rapidly, and elasticity decreases. Then, the stress was kept at 0.02 Pa at a frequency of 0.01–1 Hz under 30 °C to scan the gel and investigate G′ and G″ variation with frequency. If the G′ and G″ are independent of the shear frequency, the VES-fluid is considered solid-like; whereas if G′ and G″ are dependent of the shear frequency, the VES-fluid is considered liquid-like [33]. As seen from Figure 10b, the G′ and G″ increased slightly with the increase of the shear frequency. Consequently, the VES-fluid showed a liquid-like behavior. Furthermore, the elastic modulus of the gel was G′ > G″, indicating that the fluid mainly behaves as elastic. This may be because the storage modulus is usually related to the density of micelles, indicating the degree of entanglement of the three-dimensional micelle network [34]. Wu et al. [29] obtained similar conclusions when compounding erucamidopropyl dimethylamine with NaSal, but its G′ and G″ were higher, which may be related to the type of surfactant. Furthermore, the fluid flow properties may also be altered in response to a change in stimuli, such as temperature, salinity, and pH. In addition, in this study, as the frequency increased, the elastic modulus of the gel had certain advantages, such as viscoelastic characteristics. The viscoelasticity of the system is usually attributed to the micellar growth and the transient network formed by the entangling of wormlike micelles [29].

### 3.6. Proppants Suspending Performance

**Figure 11:**
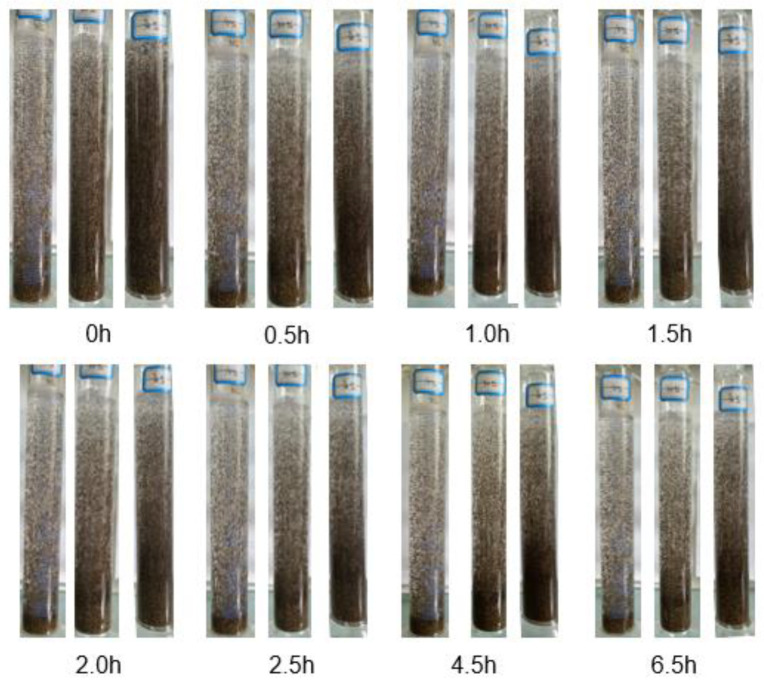
Proppants suspending performance (27 °C, 2% EAPB + 0.5% OAPB + 0.1% KCl).

The sand-carrying performance is an important index to evaluate the property of fracturing fluid. In this experiment, the proppant particles (Figure 11) with a density of 2.75 g cm^−3^ and particle size of 0.45 mm were employed to conduct the proppant suspending performance test. The content of the proppant particles in the optimized VES-based fracturing fluid was set at 10, 20, and 30%, respectively. The process of the proppant settling is shown in Figure 9. The settling velocity of the proppants in the VES-based fracturing fluid containing 10%, 20%, and 30% proppants were 1.15, 1.31, and 1.89 cm h^−1^, respectively.

### 3.7. Gel-Breaking Properties

The viscosity of viscoelastic surfactants will decrease rapidly when they encounter hydrocarbon materials [35], which is called gel breaking. Therefore, it is necessary to rate the gel-breaking properties of the optimized VES-based fracturing fluids before field tests. Table 1 shows that at 6.0% crude oil, the viscosity could be reduced to <5 mPa·s at 60 °C within 1 h. When the fracturing fluid is in contact with hydrocarbons, the micelles can encapsulate hydrocarbon molecules. For this reason, the wormlike micelles can be decomposed into many single spherical micelles, which cannot effectively enhance the viscosity of the fracturing fluid; the viscosity of the solution would be reduced rapidly [36].

### 3.8. Surface Tension

**Figure 12:**
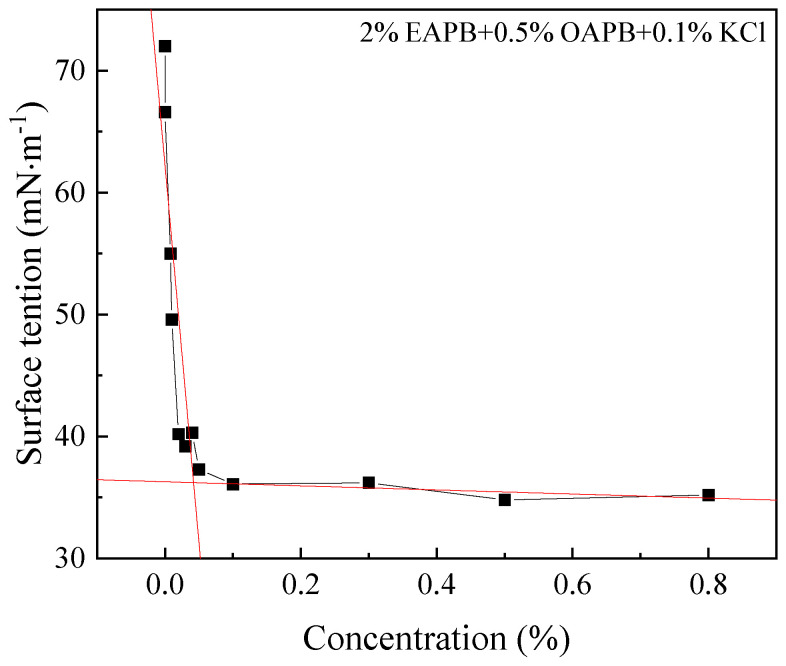
Surface tension of VES-based fracturing fluid at room temperature (27 °C).

The 2% EAPB + 0.5% OAPB + 0.1% KCl mixture solution was diluted to different concentrations, and the change in concentration of EAPB was used as the diluted solution concentration. As can be seen from Figure 12, the critical micelle concentration of viscoelastic surfactant gels was about 0.042 wt% after gel breakage. The surface tension of the viscoelastic surfactant gel decreased rapidly with increasing concentration when the solution concentration was below 0.042 wt%. However, when the solution concentration was above 0.042 wt%, the surface tension remained essentially constant. This is because the molecules can arrange on the surface and reduce the surface tension of the solution until a critical micelle concentration is reached. The surfactant molecules aggregate in the solution to form micelles and no longer change the surface tension of the solution [37].

### 3.9. Oil Displacement

**Figure 13:**
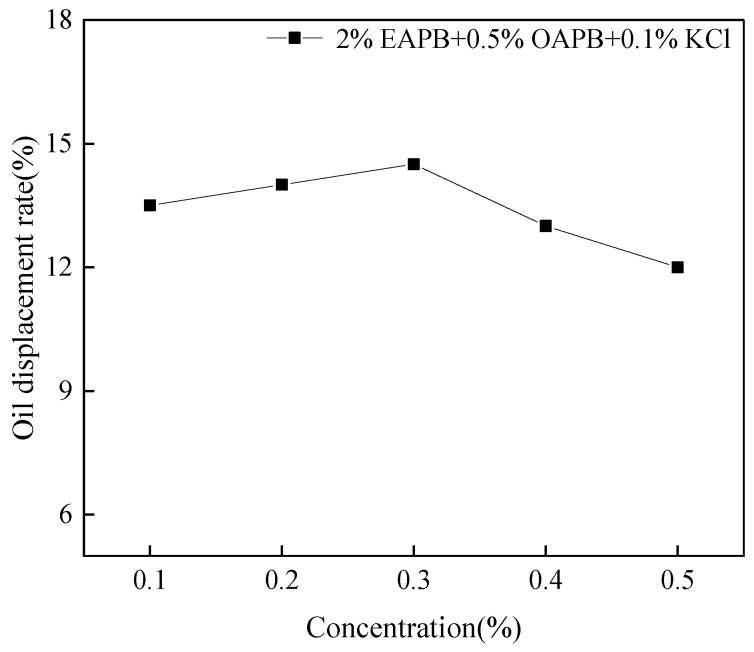
Oil displacement effect of surfactant gel.

It can be seen from Figure 13 that at the same temperature, the static oil discharge rate of the broken VES-based fracturing fluid increases first and then decreases with the increase of concentration. When the concentration was 0.3%, the oil displacement rate of viscoelastic surfactant gel increased by 14.5%. Although this is inconsistent with the conclusion that the oil drive efficiency increases first and then remains constant, the result also indicates that this viscoelastic surfactant gel improves the interfacial chemical properties of oil and water and then improves microscopic oil-washing efficiency [38]. This is due to the mobility-controlling effect of surfactants that can enter oil-containing pores that have not been swept by water and promote the growth and collision of oil droplets through emulsification and wetting inversion. Once this collision process generates enough energy to overcome the repulsive energy between oil droplets, these droplets become oil bands, thus increasing the displacement rate of static oil [17].

## 4. Conclusions

In this paper, to provide an environmentally friendly fracturing fluid formula for low permeability oilfield development, a viscoelastic surfactant gel containing 2% EAPB, 0.5% OAPB, and 0.1% KCl was prepared. The performance evaluation showed that the presence of KCl may improve the temperature resistance and increase the viscosity of the optimized fracturing fluid. Furthermore, the viscoelastic surfactant gel had high shear resistance and high sand-carrying performance. The broken gel solution was used in oil displacement tests, and the results showed that the oil displacement rate could reach 14.5% with a concentration of 0.3%.

## Figures and Tables

**Table 1:** Gel-breaking property of the VES-based fracturing fluid (2% EAPB + 0.5% OAPB + 0.1% KCl) at 60 °C.

Content of Crude Oil/wt%	Viscosity of Gel-Breaking Fracturing Fluid/mPa·s			
	1 h	2 h	2.5 h	3 h
100	2.98			
4	29.79	11.92	8.94	5.96
5	20.86	7.45	5.96	5.96
6	5.96	5.96	5.96	5.96

## Data Availability

The data presented in this study are available from the corresponding author upon request.
